# Identifying characteristics for a cost-effective psoriatic arthritis biomarker test: a development-focused health technology assessment

**DOI:** 10.1017/S0266462325000091

**Published:** 2025-04-23

**Authors:** Alexander C.T. Tam, Vinod Chandran, Dafna Gladman, Vathany Kulasingam, Eldon Spackman, Nick Bansback

**Affiliations:** 1 Centre for Advancing Health Outcomes, Providence Research, St. Paul’s Hospital, Vancouver, BC, Canada; 2 Krembil Research Institute, Schroeder Arthritis Institute, Toronto, ON, Canada; 3 Division of Rheumatology, Department of Medicine, University of Toronto, Toronto, ON, Canada; 4 Institute of Medical Science, University of Toronto, Toronto, ON, Canada; 5 Department of Laboratory Medicine and Pathobiology, University of Toronto, Toronto, ON, Canada; 6 Laboratory Medicine Program, University Health Network, Toronto, ON, Canada; 7 Community Health Sciences, University of Calgary, Calgary, AB, Canada; 8 School of Population and Public Health, Faculty of Medicine, University of British Columbia, Vancouver, BC, Canada

**Keywords:** biomarkers, technology assessment, biomedical, arthritis, psoriatic

## Abstract

**Objectives:**

This study aimed to evaluate the required test characteristics that a psoriatic arthritis (PsA) biomarker test would need to achieve to be considered cost-effective.

**Methods:**

We adapted an existing Markov model to compare a hypothetical biomarker with current practice. The model followed a patient cohort aged 45 years with moderate psoriasis (PsO) in which PsA was prevalent but unrecognized over a 40-year time horizon. Patients were assumed to be routinely seen at a dermatology clinic. In the current practice arm, patients with PsA were clinically detected. In the biomarker arm, a hypothetical test was assumed to be administered at baseline. Patients who screened positive would accept a combination of conventional disease-modifying antirheumatic drugs and targeted treatment to slow disease progression. Progression was modeled as linear changes in Health Assessment Questionnaire (HAQ) scores. We varied the sensitivity, specificity, and biomarker price based on current development progress. Scenario analyses considered alternative patient cohorts with mild and severe PsO separately.

**Results:**

The base case showed that a biomarker test with 70 percent sensitivity, 80 percent specificity, and a price of US$500 would be cost-effective (incremental cost-effectiveness ratio US$47,566 per quality-adjusted life-year [QALY]). Three-way analyses showed that a test with 80 percent specificity could be cost-effective at a US$50,000 per QALY threshold with a sensitivity as low as 66 percent at US$500. Only a near-perfect test would be cost-effective at a US$1,000 price point. Results were sensitive to HAQ progression under treatment, therapy costs, and the patient population.

**Conclusion:**

This study supports the continued product development of candidate PsA biomarkers.

## Introduction

Psoriatic arthritis (PsA) is characterized as an inflammatory arthritis that has a heterogeneous clinical presentation. Symptoms of PsA include joint pain or swelling, back pain and stiffness, nail pitting or separation, swelling of whole fingers or toes, and pain in the entheses (1). Importantly, a key clinical feature of PsA is that it commonly occurs after the onset of the skin condition, psoriasis (PsO). Because of the diversity of the symptoms that resemble common musculoskeletal symptoms and the lack of biomarkers, PsA is difficult to diagnose; the diagnosis requires the identification of inflammatory musculoskeletal disease in the context of PsO – a task that is best done by a rheumatologist (2;3). In North America, estimates suggest that PsA affects 64 per 100,000 adults (4). Estimates using claims data from private healthcare coverage employer plans suggest that the all-cause healthcare cost among PsA patients is US$29,742 per patient per year (2019 US$) (5). The burden of disease is greater among patients with PsO, affecting nearly 30 percent of patients (6–8), leading to reduced quality of life (9), greater functional impairment (10), and increased absenteeism compared to PsO alone (11). The path to the eventual diagnosis of PsA is protracted by an often lengthy period of subclinical disease that may inadvertently be controlled by certain treatments for PsO (12;13). Studies of interventions in early PsA provide evidence suggesting that intervention may improve disease activity remission (8;14), pain (15), and fatigue (14). Enabling earlier treatment through earlier detection of PsA is associated with improved treatment response and outcomes (8). The term “window of opportunity” or “window to treat” is used in rheumatology to describe a period, early in the disease course soon after disease initiation, when earlier treatment may have a long-term impact on the patient’s disease trajectory (16). If treatment is delayed beyond this window, the disease is less likely to go into remission – even with treatment – and irreversible bone erosions can occur, limiting treatment effectiveness (8;16). Therefore, there is a strong interest in identifying PsA during its subclinical phase to enable earlier intervention before this window to treat is lost (16).

Potential treatment algorithms following the early identification of PsA may include switching to conventional and targeted disease-modifying antirheumatic drugs (tDMARD), such as tumor necrosis factor inhibitors, interleukin-17 inhibitors, interleukin-23 inhibitors, and Janus kinase inhibitors, with the aim of relieving symptoms and slowing or preventing disease progression (17). However, earlier intervention needs to be balanced with increased risks of side effects, long-term efficacy, and costs borne by the patient (17). Thus, there is a need for an accurate diagnostic test for PsA to ensure that both the window of opportunity to treat is not missed (16) and the treatment is only provided to those who have undetected PsA. Patients with PsO (who are at risk for developing PsA) are usually treated by dermatologists or general practitioners (GPs) depending on the severity of PsO. Therefore, screening for PsA in dermatology and primary care clinics may facilitate early diagnosis of PsA. To date, there are several questionnaires that dermatologists can use to screen for PsA among their PsO patients (10). While cost-effective compared to no screening (18), these tools are limited by their somewhat lower sensitivity and specificity (18;19). Recently, attention has been given to the development of biomarker tests, though none have been clinically validated to date (6;20). The identification of biomarkers could help advance the management of PsA by enabling physicians to identify patients at risk of developing PsA as well as to predict treatment response, both of which have been identified as priority research areas by the Group for Research and Assessment of Psoriasis and Psoriatic Arthritis (GRAPPA) (21;22). The ability to risk-stratify patients more accurately than at present will help improve regular screening, closer monitoring of those at risk, and timely, earlier treatment within the window of opportunity (22). Candidate biomarkers include C-reactive protein, C-X-C motif chemokine ligand 10, cathelicidin LL37, and melanocytic ADAMTSL5 autoantibodies (6;20), with evidence suggesting that panels of multiple markers are a promising path forward (6).

The development of biomarkers is a costly process, fraught with uncertainties and risks (23). A test is ultimately only successful if it is used – not just because of its clinical utility, but also due to its economic value, which leads to reimbursement by payors (24). While reimbursement is ultimately guided by health technology assessment (HTA) at the end stage of product development, early HTA (eHTA) and development-focused HTA can provide an indication of a technology’s potential to be considered cost-effective (25). The use of early economic models can guide developers in deciding whether to continue developing the biomarker, revisit other candidate biomarkers or technologies, or cease development altogether, along with decisions around pricing and informing pricing expectations (26;27). Therefore, the objective of this study was to identify the necessary performance metrics (i.e., diagnostic specificity and diagnostic sensitivity) that potential biomarkers for PsA would need to achieve, at a range of potential competitive prices for these biomarker tests that would be considered cost-effective by payors in the United States. This study will directly inform a separate research team led by V.C. (project title: *Multi-Omic Diagnostic Test for Psoriatic Arthritis in Psoriasis Patients*) that is currently developing biomarkers for PsA (28). The study should also help other biomarker test developers understand the economic viability of their candidate products.

## Methods

### Study overview

This study reports on an economic model we developed as a part of an eHTA for a PsA biomarker test that is being developed. We further developed an existing Markov model (18), which had been developed to evaluate screening questionnaires for PsA. The choice of a Markov model was because the existing model was readily available for us to adapt, and the data that we had identified in the literature were well suited for the adapted Markov model. Thus, it was for practical reasons that we chose the Markov model. Guided by a team developing biomarkers for PsA, we considered a range of plausible prices and performance metrics (i.e., diagnostic sensitivity and diagnostic specificity) of a hypothetical biomarker test and compared costs and quality-adjusted life-years (QALYs) with those of no screening. The development team (led by V.C.) has extensive experience with engaging regulatory bodies and payors and had predetermined *a priori* that performance, prices, and an indication of cost-effectiveness were important inputs or metrics for past and ongoing discussions about coverage and reimbursement. A U.S. payor perspective was taken, and reporting followed the Consolidated Health Economic Evaluation Reporting Standards guidelines (29). Costs and QALYs were discounted by 3 percent in line with guidance for economic evaluations conducted from the U.S. perspective (30;31). Research Ethics Board review was not required for this study because it relied solely on data obtained from published research literature.

### Model structure

The existing model considers patients with mild PsO who undergo routine screening using questionnaires in annual cycles (18). PsO patients have a chance to develop PsA that is initially undetectable by the screening tools. Disease progression is represented by linear changes in Health Assessment Questionnaire (HAQ) scores. We have used the HAQ as a proxy for tracking disease progression for the purpose of setting points within the model where patients may experience different events once HAQ scores exceed thresholds (Table 1) (18;49). This is a common approach in inflammatory arthritis economic models (50). The HAQ is not a part of the intervention.

**Table 1.** Model parameters

Parameter	Base case value	Standard error for probabilistic analyses	Range for deterministic analyses	Source
*Disease progression, probabilities*				
Prevalence of undetected PsA among PsO patients	12.38%	Fixed	Fixed	(32;33)
Baseline HAQ	0.5	Fixed	Fixed	Assumption
HAQ progression − no treatment	+0.077	0.03, Gamma	±25%	(34)
HAQ progression − on MTX	+0.032	0.003, Gamma	±25%	(34)
HAQ progression − on combination cDMARD	+0.020	0.010, Gamma	±25%	Assumption, coefficient of variation = 0.5
HAQ progression − on tDMARD	+0.000	0.02, Gamma	±25%	(34)
HAQ progression − difficult to treat	+0.078	0.03, Gamma	±25%	(35)
HAQ reduction − tDMARD initiation	−0.417	Triangular	−0.522, −0.280	(34)
HAQ rebound − tDMARD loss of effect	+0.417	Triangular	+0.522, +0.280	(34)
Probability of infection	0.052	0.002, Beta	±25%	(36)
Probability of malignancy	0.005	0.003, Beta	±25%	(37)
Mean time on tDMARD (years)	5	Fixed	Fixed	(18;38)
Probability of accepting treatment plan	100%	Fixed	Fixed	Assumption
*Utilities*				
Baseline utility	0.884	0.01, Normal	±25%	(34)
Utility and HAQ linear correlation	−0.259	0.009, Normal	−0.295, −0.232	(34)
Utility and PASI linear correlation	−0.003	0.001, Normal	−0.004, −0.002	(34)
Disutility of infection	−0.012	Triangular	±25%	(18;39)
Disutility of malignancy	−0.05	0.019, Normal	±25%	(40)
*Costs*				
Price of biomarker test	US$500, US$1,000, US$1,500	Fixed	Fixed	Assumption
Rheumatologic assessment	US$906.52	8.75, Gamma	±25%	(41;42)
Topical treatment for PsO	US$1,149.69	333.10, Gamma	±25%	(43;44;45)
MTX	US$126.88	24.38, Gamma	±25%	(43;44;45)
cDMARD combination	US$5,832.07	3,665.06, Gamma	±25%	(43;44;45)
TNF inhibitor	US$51,896.15	6,558.83, Gamma		
IL–17 inhibitor	US$86,628.89	8,879.76, Gamma	±25%	(43;44;45)
IL–23 inhibitor	US$81,976.48	5,078.17, Gamma	±25%	(43;44;45)
JAK inhibitor	US$36,309.16	9,418.72, Gamma	±25%	(43;44;45)
Infection	US$5,873.00	2,936.5, Gamma	±25%	(46)
Malignancy	US$14,868.87	11,255.29, Gamma	±25%	(47)
Direct medical cost at HAQ 0	US$4,238	Fixed	Fixed	(48)
Direct medical cost per 1 HAQ gain	1.38 times	0.16, Normal	±25%	(48)

cDMARD, conventional disease-modifying antirheumatic drug; HAQ, Health Assessment Questionnaire; MTX, methotrexate; PASI, Psoriasis Area and Severity Index; PsA, psoriatic arthritis; PsO, psoriasis.

In our adaptation, we consider PsO patient populations where PsA is already prevalent but undetected. They will be detected clinically once PsA symptoms become evident to the patient, and they present to the clinic with their concerns (see the “Disease Progression” section for how this was operationalized). They are referred for a rheumatologic assessment, and once diagnosed, they will receive treatment. We consider the addition of a biomarker test used at model entry, with those testing positive transitioning to treatment for PsA (“early treatment”) and those testing negative continuing to be treated for their PsO until clinical detection. Patients progress through the model in annual cycles until death or the end of a 40-year time horizon, in line with the existing model (18). The adapted model was developed with input from a clinical expert on PsA (V.C.).

### Study population

We considered three populations in which a biomarker test may be used: mild PsO seen at a GP’s office, moderate PsO seen at a dermatology clinic, and severe PsO seen at a dermatology clinic. The moderate PsO population served as our base case in which a biomarker test will see the most clinical use according to expert opinion. We considered a population of patients with moderate PsO as defined by a Psoriasis Area and Severity Index (PASI) score of 6 and aged 45 years at baseline. PsO is assumed to be adequately managed by methotrexate. Patients present to their usual dermatology clinic (or, in the case of the population with mild PsO, to their usual GP’s office).

#### No-test standard of care pathway

Patients in the no-test arm are routinely followed in the dermatology clinic for their PsO. Following clinical detection of PsA, they continue treatment with methotrexate. Once the disease progresses even further, they will switch to treatment with tDMARDs. Eventually, patients may reach a state where the disease is considered “difficult to treat,” and they will remain in this state until the end of the model or death.

#### Suggested new biomarker pathway

**Figure 1.**
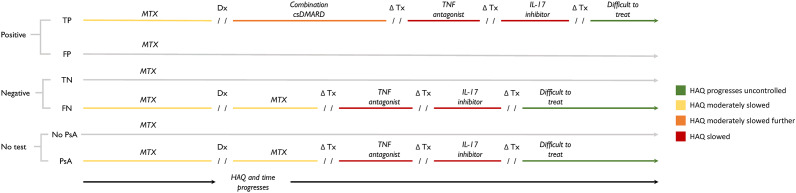
Treatment consequences result from testing positive (top), testing negative (middle), and no testing (bottom). Test results (positive or negative) are used to guide the subsequent treatment pathway of patients. Patients who test positive will continue using methotrexate until PsA develops to the point of clinical detection. Once diagnosed, test-positive patients switch to combination conventional DMARD therapy, which is intended to slow PsA progression to a greater degree than monotherapy methotrexate, thereby delaying eventual initiation of tDMARDs. Abbreviations: Dx, diagnosis; FN, false negative; FP, false positive; HAQ, Health Assessment Questionnaire; PsA, psoriatic arthritis; TN, true negative; TP, true positive; Tx, treatment.

In this pathway, we considered a hypothetical biomarker test that is an add-on. We assume that this add-on test would be used by a physician to help detect PsA. We currently do not know what the analyte the hypothetical test would be detecting is. The biomarker test is administered at baseline. Positive results are followed up with an assessment by a rheumatologist, and a treatment plan is recommended (Figure 1). Treatment pathways were based on expert opinion and partially supplemented by reference to the GRAPPA treatment recommendations (51). To facilitate expert consultation, we created three patient vignettes and treatment plans that corresponded to the three target populations of our model. We asked two experts to review the vignettes independently and to provide written responses to questions about the appropriateness of the treatment pathway, including alternative treatment pathways and the order of specific treatments. For test-positive patients, the subsequent treatment was combination DMARD therapy if disease progression reached the point of clinical detection. Further PsA disease progression while on combination DMARD therapy would result in a switch in treatment to the next lines of therapy: tDMARDs. False positives who initiate early treatment would receive treatment for PsA. That is, a proportion of patients who test false positive will be overtreated, although the treatment is also used for patients with moderate and severe PsO (52;53). Patients who are false negatives, like patients in the no-screening arm, are treated when their PsA symptoms and signs are present. False negatives do not experience any additional harms compared to those who are not screened.

### Disease progression

PsA disease progression was modeled using HAQ as a proxy measure as done in the existing model and others in the literature (50). Progression was reflected as annual changes in HAQ score. HAQ has a range of 0–3, with higher scores indicating worse functional ability (54). Changes in HAQ were modeled as a linear function that changed depending on the treatment as reported in the literature (Table 1) (34;35). We assumed that clinical detection would occur in the year that undetected PsA progressed to a HAQ score of 0.71, which corresponds to the mean HAQ score in a study of patients referred to a clinic for early arthritis presenting with symptoms associated with PsA (18;49). Treatment with a conventional disease-modifying antirheumatic drug (cDMARD) would slow HAQ progression relative to no treatment; treatment with a tDMARD would reduce HAQ initially, but loss-of-effect was assumed to occur after 5 years where HAQ bounces back by the initial reduction; patients who enter the difficult-to-treat state would have a steeper increase in HAQ relative to no treatment (34;35). We assumed that the switch from combination cDMARD therapy to tDMARDs (in the case of true positives) and from cDMARD therapy to tDMARDs (in the case of false negatives and patients in the no-screening arm) would occur in the year that HAQ score reached 1.05, which corresponds to the mean HAQ score derived from six clinical trials that examined clinical effectiveness of etanercept, infliximab, and adalimumab among patients with active PsA who had inadequate response to conventional therapy (18;34). The impact of HAQ thresholds on model outcomes was assessed in sensitivity analyses. For example, by lowering the HAQ threshold at which tDMARD initiation starts, the patients who continue to experience progression will be treated earlier in their disease course than if the HAQ threshold for treatment was higher. This earlier treatment with tDMARD therapy would result in patients spending fewer model cycles on cDMARD therapy and more cycles in the “difficult-to-treat” state later in the model, both of which affect the accumulation of costs and utility compared to a model with different HAQ score thresholds.

PsA is prevalent and undetected; therefore, a positive HAQ was assumed at baseline (55). Modeled HAQ scores were constrained to the range of 0–3 using a function to limit scores if the increase or decrease in scores would result in an impossible HAQ score. The severity of the skin component of the disease, measured by PASI, was assumed to be constant in line with the previous model (18). The mortality rate of patients with PsA was assumed not to differ from that of the general population (18); thus, age-specific probabilities were obtained from U.S. life tables (56).

### Quality of life

HAQ scores and baseline PASI scores were converted to health utility values (scale of 0 = death to 1 = perfect health) according to average linear correlations previously reported between the EQ-5D versus the two scores (18;34). A 1-unit change in HAQ was associated with a decrease in utility of 0.259 (18;34). QALYs were calculated by multiplying the health utility values with the time spent at each HAQ level. The use of tDMARDs has been associated with an increased risk of certain adverse events; therefore, we also considered disutilities and probabilities associated with serious infections requiring hospitalization or intravenous antibiotic treatment, or those leading to death, as well as the development of any malignancies (such as skin cancers) while on tDMARD, which were obtained from the literature (18;36;37;39).

### Cost

Direct costs included office visits and diagnostic procedures (e.g., biofluid tests and imaging studies) for rheumatologic assessments, direct medical costs associated with changes in HAQ, drugs, costs associated with malignancies and infection, and the price of a biomarker test. Costs of physician time and procedures were obtained from CMS physician fee schedules and clinical laboratory fee schedule files, respectively (41;42). Direct medical costs associated with HAQ progression were derived from a published study using data from the National Databank for Rheumatic Diseases in the United States (48). Annual costs related to adverse events were obtained from the literature (47;46). The midpoint between the National Average Drug Acquisition Cost (NADAC) and the Veterans Affairs Federal Supply Schedule (VAFSS) cost for drugs was used as described elsewhere (43). Levy et al. (43) found that Wholesale Acquisition Cost generally overstates drug costs in the United States, the discount applied to WAC is highly variable, and their use of both NADAC and VAFSS is an assumedly closer approximation of what payors are likely to pay (43). Costs were inflated to 2021 US$ using the medical care component of the U.S. consumer price index (57).

### Analysis

We followed a hypothetical cohort of 1,000 patients through both strategies over a 40-year period until death or the end of the time horizon. We calculated the total cost and QALY for the cohort for different pairs of sensitivity and specificity across a range of potential prices for a biomarker test. We calculated the mean cost and QALYs per patient, and the incremental cost-effectiveness ratio (ICER) and net monetary benefit were computed by comparing biomarkers against no screening. Base case results are presented as multiway sensitivity analyses where sensitivity, specificity, and price are varied. As a base scenario, an arbitrary combination was selected: The test was assumed to have a sensitivity of 70 percent, a specificity of 80 percent, and a price of US$500. The willingness-to-pay threshold was set at US$50,000 per QALY.

As scenario analyses, we modeled the mild and severe PsO populations in which we varied the baseline PsO treatment, prevalence of undetected PsA, proportion of positive tests that accept early treatment, and the treatment plan recommended (Supplementary Figure S1 and Supplementary Table S1).

We selected the moderate PsO patient population to further conduct deterministic and probabilistic sensitivity analyses, given that this population is likely where a biomarker test will see the most clinical use, according to expert opinion. Model parameters were varied as defined in Table 1. For the probabilistic sensitivity analysis, 10,000 simulations were run by drawing inputs from appropriate probability distributions. Sensitivity, specificity, and costs were fixed in probabilistic analyses. Our model was developed and analyzed using Microsoft Excel^®^ live version 365.

## Results

### Base population – biomarker test used in patients with moderate PsO who are routinely followed at a dermatology clinic

As a base case, the test was assumed to have a sensitivity of 70 percent, a specificity of 80 percent, and a price of US$500. The mean cost and QALY of using a biomarker test (US$50,792, 17.20 QALYs) were higher than if no test were used (US$49,975, 17.18 QALYs). The higher cost is attributable to the increased cost of treatment for test-positive patients, whereas the higher mean QALYs are associated with delayed tDMARD usage among true positives as a result of slowed progression from combination therapy. The consequent ICER is US$47,566 per QALY (95 percent confidence interval = − US$1,456,697, US$922,661). Among replicates where the estimated ICER was negative, 82 percent replicates were due to the biomarker having lower costs and higher QALYs compared to no screening (biomarker was dominant) and 18 percent were due to the biomarker having higher costs and lower QALYs compared to no screening (biomarker was dominated). The main drivers of the lower QALYs in dominated replicates were HAQ progression under methotrexate being lower (i.e., patients in the no-screening arm would have inadvertently better-controlled disease progression, diminishing the value of testing and treating early) and HAQ progression in the difficult-to-treat state being lower (i.e., the benefit of spending fewer cycles in this state by controlling disease earlier was diminished).

**Figure 2.**
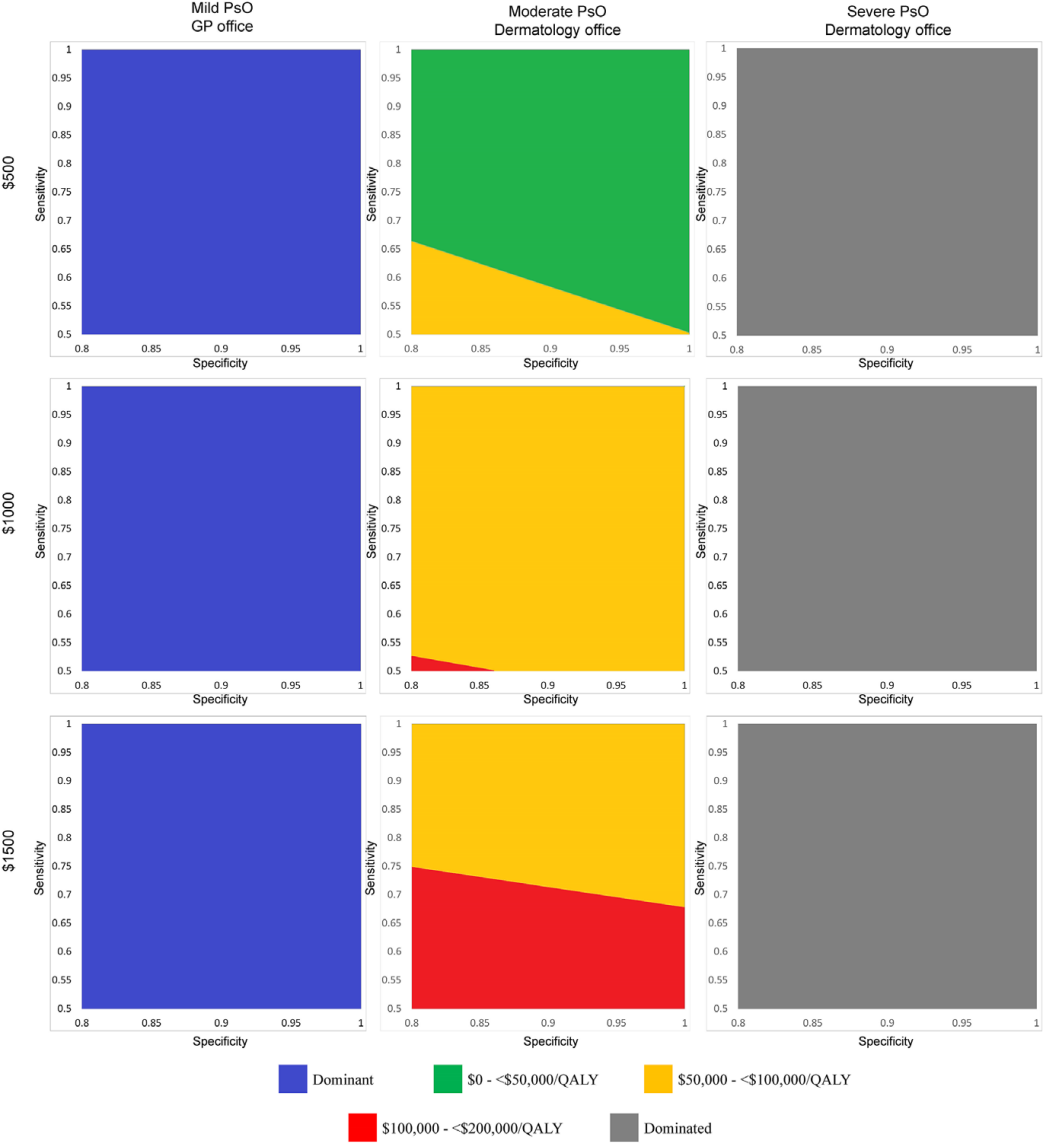
Two-way sensitivity analysis plots showing incremental cost-effectiveness ratios for combinations of sensitivity and specificity at three price points for a hypothetical biomarker test used in three patient populations. Each graph corresponds to a combination of price point (first row: US$500; second row: US$1,000; third row: US$1,500) and targets patient population (first column: mild PsO, biomarker used in the GP setting; second column: moderate PsO, biomarker used in the dermatology office setting; third column: severe PsO, biomarker used in the dermatology office setting). For each price and target patient population combination, we reran the model using different pairs of sensitivity and specificity as inputs to calculate a range of ICERs. We categorized each ICER according to different ranges of willingness-to-pay thresholds and plotted these as regions on each graph. For example, in the moderate PsO population and US$500 price point combination (first row, second column), at a specificity of 0.80, the minimum sensitivity that a biomarker test would need to achieve to have an ICER of <US$50,000/QALY gained would be 0.66. At a specificity of 0.90 in the same graph, the minimum sensitivity would need to be 0.58. At a specificity of 0.95 in the same graph, the minimum sensitivity could fall to 0.54 and the ICER would still remain <US$50,000/QALY gained. Abbreviations: GP, general practitioner; ICER, incremental cost-effectiveness ratio; PsO, psoriasis; QALY, quality-adjusted life-year.

In multiway analyses of the impact of sensitivity, specificity, and price combinations, the ICER comparing biomarker to no screening ranged from US$29,595 (perfect test, priced at US$500) to US$144,362 (50 percent sensitivity, 80 percent specificity, US$1,500) (Table 2 and Figure 2). A perfect test could potentially be priced up to US$1,000, and the resulting ICER of US$49,960 per QALY would still be considered cost-effective with a US$50,000 per QALY willingness-to-pay threshold.

**Table 2.** High-low estimates of incremental cost-effectiveness ratios comparing a hypothetical biomarker test against no screening in three patient populations based on varied sensitivity, specificity, and test price

	Biomarker cost (US$)	No-screening cost (US$)	Biomarker QALY	No screening QALY	ICER (US$)	Sensitivity, specificity, price
*Base case*						
Mild PsO	40,438	51,701	17.59	17.51	−146,511	70%, 80%, US$500
Moderate PsO	50,792	49,975	17.20	17.18	47,566	70%, 80%, US$500
Severe PsO	985,824	985,538	16.84	16.84	Dominated	70%, 80%, US$500
*Lowest ICER*						
Mild PsO	43,212	51,701	17.56	17.51	−154,592	50%, 80%, US$500
Moderate PsO	50,702	49,975	17.20	17.18	29,595	100%, 100%, US$500
Severe PsO	985,781	985,538	16.84	16.84	Dominated	100%, 100%, US$500
*Highest ICER*						
Mild PsO	46,265	51,701	17.57	17.51	−98,991	50%, 100%, US$1,500
Moderate PsO	51,747	49,975	17.19	17.18	144,362	50%, 80%, US$1,500
Severe PsO	986,893	985,538	16.84	16.84	Dominated	50%, 80%, US$1,500

ICER, incremental cost-effectiveness ratio; PsO, psoriasis; QALY, quality-adjusted life-year.

At a sensitivity of 55 percent, the lowest corresponding specificity that a biomarker test would need to achieve would be 94 percent to be considered cost-effective at US$500 (ICER: US$49,786 per QALY) (Table 3). Increasing sensitivity decreased the required specificity for a test to be considered cost-effective. A US$500 biomarker test would be considered cost-effective at 60 percent sensitivity if it achieves a specificity of at least 88 percent (ICER: US$49,642 per QALY). Increasing the sensitivity to 70 percent would reduce the required specificity to 80 percent, which was the lowest specificity considered in the model (ICER: US$47,566 per QALY).

**Table 3.** Minimum sensitivity and specificity required for a US$500 hypothetical biomarker used among a population with moderate PsO to remain cost-effective at US$50,000/QALY according to multiway analyses

Parameter	The lowest value for other parameter	Incremental Costs (US$)	Incremental QALYs	ICER
*Specificity*	*Sensitivity*			
80%	66%	808.42	0.016	US$49,890/QALY
85%	62%	759.64	0.015	US$49,904/QALY
90%	58%	710.86	0.014	US$49,920/QALY
95%	54%	662.08	0.013	US$49,939/QALY
100%	50%^a^	613.30	0.012	US$49,960/QALY
*Sensitivity*	*Specificity*			
50%	100%	613.30	0.012	US$49,960/QALY
55%	94%	672.29	0.014	US$49,787/QALY
60%	88%	731.28	0.015	US$49,642/QALY
65%	82%	790.27	0.016	US$49,520/QALY
70%	80%^a^	817.48	0.017	US$47,566/QALY

a Values were the lowest considered for these parameters in the model.

ICER, incremental cost-effectiveness ratio; PsO, psoriasis; QALY, quality-adjusted life-year.

At the lowest specificity considered in the model (80 percent), the resulting sensitivity needed could be as low as 66 percent and a US$500 test would remain cost-effective (ICER: US$49,890 per QALY). Raising the specificity to 85 percent would reduce the required sensitivity to 62 percent (ICER: US$49,904 per QALY). At a specificity of 95 percent, the corresponding sensitivity would be 54 percent (ICER: US$49,939 per QALY). Results were sensitive to the price considered: At the US$1,000 price point, only a perfect test would be considered cost-effective; at US$1,500, no performance combination would result in a cost-effective biomarker.

One-way sensitivity analyses showed that the assumed HAQ progression while using combination cDMARD had the greatest impact on the results (Supplementary Figure S2). A progression that is 25 percent higher than the base case (i.e., progression more akin to methotrexate alone) reduces the delay of tDMARD initiation, leading to higher costs and lower QALYs. The costs of cDMARDs and tDMARDs have opposite effects from each other: A 25 percent increase in combination therapy cost raises the ICER as it imposes a significant cost on patients compared to monotherapy, whereas a 25 percent increase in tDMARD cost lowers the ICER as the years of use delayed due to combination therapy are realized as savings. Reducing the time between diagnosis and tDMARD start primarily affects the ICER by reducing the incremental QALYs.

A probabilistic sensitivity analysis was conducted assuming that the hypothetical biomarker test was administered to patients with moderate PsO, with 70 percent sensitivity, 80 percent specificity, and a price of US$500. At a willingness to pay of US$50,000/QALY, such a test would have a 58 percent probability of being cost-effective compared to no screening (Supplementary Figure S3).

### Alternate population – biomarker test used in patients with mild PsO who are routinely followed in primary care

Assuming the same base case combination as in the previous population (70 percent sensitivity, 80 percent specificity, and a price of US$500), using a biomarker test in patients with mild PsO would result in a mean per patient cost of US$40,438 for 17.59 QALYs gained. In the no-screening arm, the costs and QALYs were US$51,701 and 17.51, respectively. Since the biomarker yielded negative incremental costs and positive incremental QALYs (ICER: −US$146,511 per QALY), the use of a biomarker test would be considered cost-saving and a dominant strategy over no screening. This can be attributed to the treatment pathway modeled for this population: Test-positive patients are immediately switched onto methotrexate, whereas test-negative and no-screening patients are maintained on topical treatment for PsO until clinical detection, which assumedly does not control nonskin components of PsA. The combination of significantly earlier treatment of true positives and proportionally fewer patients who wait until PsA reaches clinical stages reduces future treatment costs and improves HAQ progression relative to no screening. A biomarker test would be considered cost-saving under all combinations of sensitivity, specificity, and price points (lower ICER: −US$154,592 per QALY; upper ICER: −US$98,991 per QALY) (Table 2).

### Alternate population – biomarker test used in patients with severe PsO who are routinely followed at a dermatology clinic

In contrast to results from the mild PsO scenario, using a biomarker test in patients with severe PsO would result in higher costs (US$985,824) compared to no screening (US$985,538) over 40 years. Incremental QALYs were zero (16.84 QALYs in both arms). The treatment pathway had a significant role in this result as well. Patients with severe PsO were already established on a tDMARD at baseline. Patients continued to use the initial tDMARD before switching as a result of loss of effect and neither the initial tDMARD nor the time to switch was influenced by screening results. The consequent ICER could not be calculated because the denominator was zero. The biomarker arm was dominated by no screening – that is, it resulted in no QALY gain and higher costs – under all combinations of sensitivity, specificity, and price considered.

## Discussion

We analyzed the cost-effectiveness of a hypothetical biomarker test to identify undetected PsA among patients with PsO. We find that at a price of US$500, a biomarker test with sensitivity and specificity above 70 and 80 percent may potentially provide reasonable value for money in patients with moderate PsO who are routinely followed in a dermatology clinic and could be reimbursed. The test would also potentially be cost-effective at US$50,000 per QALY threshold in a larger market of patients being seen in general practice, but the implementation of such a test would be more challenging. It is unlikely that such a test would be of value in patients with severe PsO.

To the best of our knowledge, the only other model that assesses a tool for early PsA detection is the model that we adapted from Iragorri et al. (18). The tools considered were screening questionnaires and were used annually to detect PsA among patients with mild PsO during an assumed 2-year window before normal detection. The Early Psoriatic Arthritis Screening Questionnaire, the Toronto Psoriatic Arthritis Screen, and the Psoriasis Epidemiology Screening Tool were all considered to be cost-saving relative to no screening. The result is similar to findings in our alternate patient population – patients with mild PsO who are seen by their GP. Further, the direction of change in ICER from using tDMARD as first-line therapy and reduced effectiveness of cDMARDs in Iragorri et al. (18) is similar to our one-way sensitivity analyses. However, the cost-saving nature of a biomarker test among patients with mild PsO seen by their regular GP should be read with caution. Implementing a testing program in this population would require an additional layer of healthcare coordination to ensure timely referral from primary care to dermatology and rheumatology (58). The lack of experience and knowledge about PsA, its long-term impact, and associated consequences impedes developing a screening and referral strategy, with or without a biomarker-based test (59). There is a lack of collaboration among GPs, dermatologists, and rheumatologists aimed at early PsA diagnosis in most healthcare settings (59). Future studies should consider how screening in primary care can exploit the potential cost savings suggested by this study. At present, since there is better collaboration between dermatologists and rheumatologists (60), the use of screening questionnaires (18) or a biomarker test in the dermatology clinic setting may prove to be a feasible option.

One eHTA approach to examine the early diagnosis of rheumatoid arthritis (RA) was explored by Buisman et al. (61), who examined four potential diagnostic tests: B-cell RNA expression, IL-6 serum level test, MRI, and a genetic assay with susceptibility single nucleotide polymorphisms, all against the RA-2010 criteria. Each test had an assumed sensitivity, specificity, and price elicited from test developers and the literature. As an add-on test, B-cell RNA expression (60 percent sensitivity and 90 percent specificity) was considered a cost-effective strategy if it were used for all patients, while other tests were not, primarily due to their lower specificity (61). The authors further calculated that using a B-cell RNA expression test has a maximum headroom of €511 compared to expected test costs of €150 if it were used in patients at intermediate risk of RA. While our headroom analysis only considered discrete price points, our results would suggest that a biomarker test for PsA could be priced at US$500 and potentially higher with a ceiling of US$1,000 depending on test performance.

The present application of economic modeling to inform the requisite diagnostic sensitivity and specificity for a hypothetical biomarker test situates itself within a broader field of using early economic evaluations to inform Target Product Profiles (TPP) – the necessary properties of a new product to meet an unmet need (62). PsA biomarker identification and test development is a critical research area in this clinical field, with clearly defined clinical needs (21;63). Our results on the combinations of sensitivity and specificity of a hypothetical test may provide a general indication or sense of potential performance metrics, but formal TPPs are still needed such as the ones set out by the World Health Organization (64). As the results of validation studies of different biomarkers become available, test developers will benefit from our results. The potential headroom should also be informative for test developers to weigh against the costs of the technologies involved in their future test panels. We also show in sensitivity analyses that, in addition to properties inherent to the biomarker test, factors related to the clinical context (i.e., treatment implications of test results) are highly important to cost-effectiveness (62).

The results should be interpreted in light of this study’s limitations. First, we made several clinical assumptions that greatly influenced the model results. The most critical clinical assumption we made was regarding the effectiveness of combination treatment given a paucity of data specific to its impact on HAQ progression. Sensitivity analyses showed that its impact on the model was greater than that of any other parameter. This was followed by the assumed timing of tDMARD initiation in patients with moderate PsO. Assuming that tDMARDs were used following any progression on cDMARDs increased the incremental costs and decreased the incremental QALYs. Second, we modeled three patient populations representing three severities of PsO; however, we did not model changes in PASI over the model time horizon (i.e., PASI was at a constant level). We assumed that the treatment received for PsA would be effective in controlling the skin component of the condition. Further, the utility–PASI correlation is much smaller than the utility–HAQ correlation; as such, we believe that it would not have changed the model results significantly. Finally, disease progression was estimated using linear changes in HAQ that were only modified by the treatment course. HAQ may be affected by other clinical and demographic variables that we could not consider without individual-level data (34). Relatedly, we followed the assumption of the existing model that all patients would experience some response to tDMARD therapy in the form of an average reduction in HAQ. This could overestimate the improvement in health utility and costs of tDMARDs and the time that patients remain on each line of tDMARD therapy, which could in turn overestimate the cost-effectiveness of the biomarker test, given that a key driver of the benefit of testing is derived from delaying tDMARD use. In one-way sensitivity analyses, we varied the reduction in HAQ due to tDMARD therapy and found that its impact was minimal and was not a key parameter for the model; rather, the slowed HAQ progression while on cDMARDs had a far greater influence on model results. One of the main strengths of this study is the use of an existing model that we independently validated, adapted, and expanded upon (18). We also modeled three distinct patient populations, including the one that we believe a future PsA biomarker test would be most practical for. While this analysis has been conducted from a U.S. perspective, we expect that similar inferences would likely be drawn in other North American and European countries regarding the key drivers of biomarker cost-effectiveness.

As a research agenda, we propose two topics to elucidate further. First, it is worth reiterating the importance of understanding the effectiveness of cDMARDs and combinations of conventional cDMARDs in slowing disease progression after early PsA detection. As Iragorri et al. (18) noted for the original model, the cost-effectiveness of, in their study, a PsA screening program and, in our study, the use of a biomarker test will hinge on how well cDMARDs can control disease progression before tDMARD use. Second, as data become increasingly available on treatment effectiveness in early PsA, additional economic modeling approaches may be considered. The heterogeneous nature of PsA and the patient’s response to therapy, as well as the lack of available data on plausible ranges of sensitivity and specificity for the hypothetical biomarker test, presented modeling challenges. Discrete event simulation models may offer a more natural approximation of disease progression, as well as a means to account for patient-level differences in response and adverse event experiences.

## Conclusion

This study showed that a biomarker test to detect PsA in patients with moderate PsO can be cost-effective if it achieves reasonable performance and is well priced. Beyond the test itself, clinicians and advocates of early detection should also consider the treatment implications of early detection as this is paramount to the reason for testing.

## Supporting information

Tam et al. supplementary material Tam et al. supplementary material

## Supplementary material

The supplementary material for this article can be found at http://doi.org/10.1017/S0266462325000091.
