# Supplementary material for: Identifying characteristics for a cost-effective psoriatic arthritis biomarker test: a development-focused health technology assessment
Source: Int J Technol Assess Health Care. 2025 Apr 23;41(1):e29. doi: 10.1017/S0266462325000091 (PMC12123161; doi:10.1017/S0266462325000091)
Supplement: Tam et al. supplementary material [file S0266462325000091sup001.docx]

Contents

[Figure S1. Alternate treatment pathways among those with mild psoriasis (panel A) and those with severe psoriasis (panel B). 2](#_Toc154070437)

[Table S1. Model parameters altered in scenario analyses. 3](#_Toc154070438)

[Figure S2. Tornado diagram showing the ten most influential model parameters in one-way sensitivity analyses. Red line represents the base case ICER. Abbreviations: MTX = methotrexate, LEF = leflunomide, HAQ = health assessment questionnaire. 4](#_Toc154070439)

[Figure S3. Cost-effectiveness acceptability curve showing the probability that the biomarker is cost-effective at various willingness-to-pay thresholds. 5](#_Toc154070440)

[REFERENCES 6](#_Toc154070441)


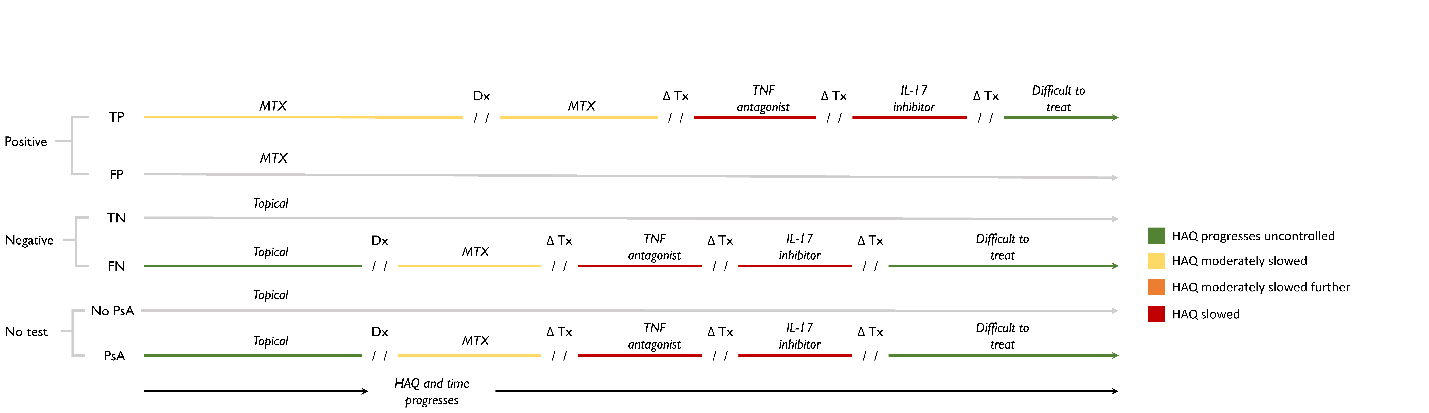


A

B


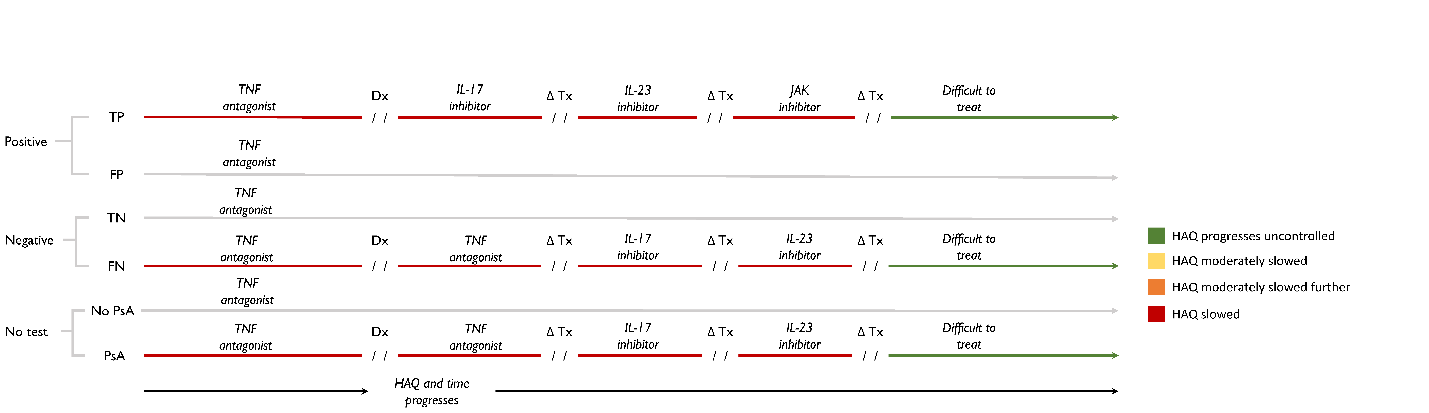


*Figure S1. Alternate treatment pathways among those with mild psoriasis (panel A) and those with severe psoriasis (panel B).* Treatment consequences that result from testing positive (top), testing negative (middle), and no testing (bottom). Abbreviations: TP = true positive, FP = false positive, TN = true negative, FN = false negative, PsA = psoriatic arthritis, Dx = diagnosis, Tx = treatment, HAQ = health assessment questionnaire.

# Table S1. Model parameters altered in scenario analyses.

|  | Mild psoriasis | Severe Psoriasis |
| --- | --- | --- |
| Prevalence | 10.1% (1) | 20.6% (2) |
| Baseline PASI | 3.5 (3) | 12 (assumption) |
| Baseline HAQ | 0 (3) | 0.5 (assumption) |
| Probability of accepting treatment change following positive test | 90% (assumption) | 95% (assumption) |
| Probability of PsO progressing | 3.1% (4) | 0% (assumption) |


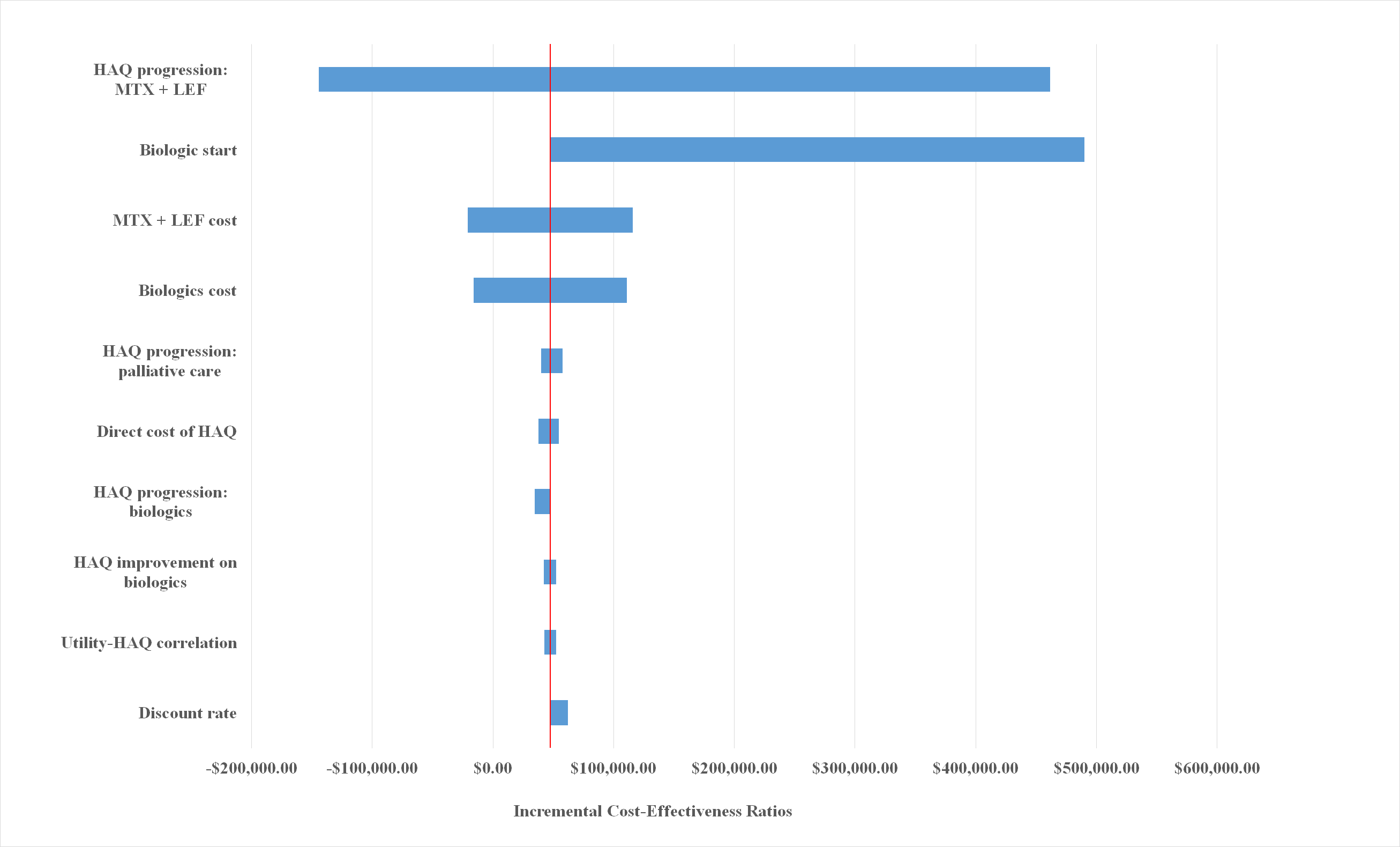


# Figure S2. Tornado diagram showing the ten most influential model parameters in one-way sensitivity analyses. Red line represents the base case ICER. Abbreviations: MTX = methotrexate, LEF = leflunomide, HAQ = health assessment questionnaire.


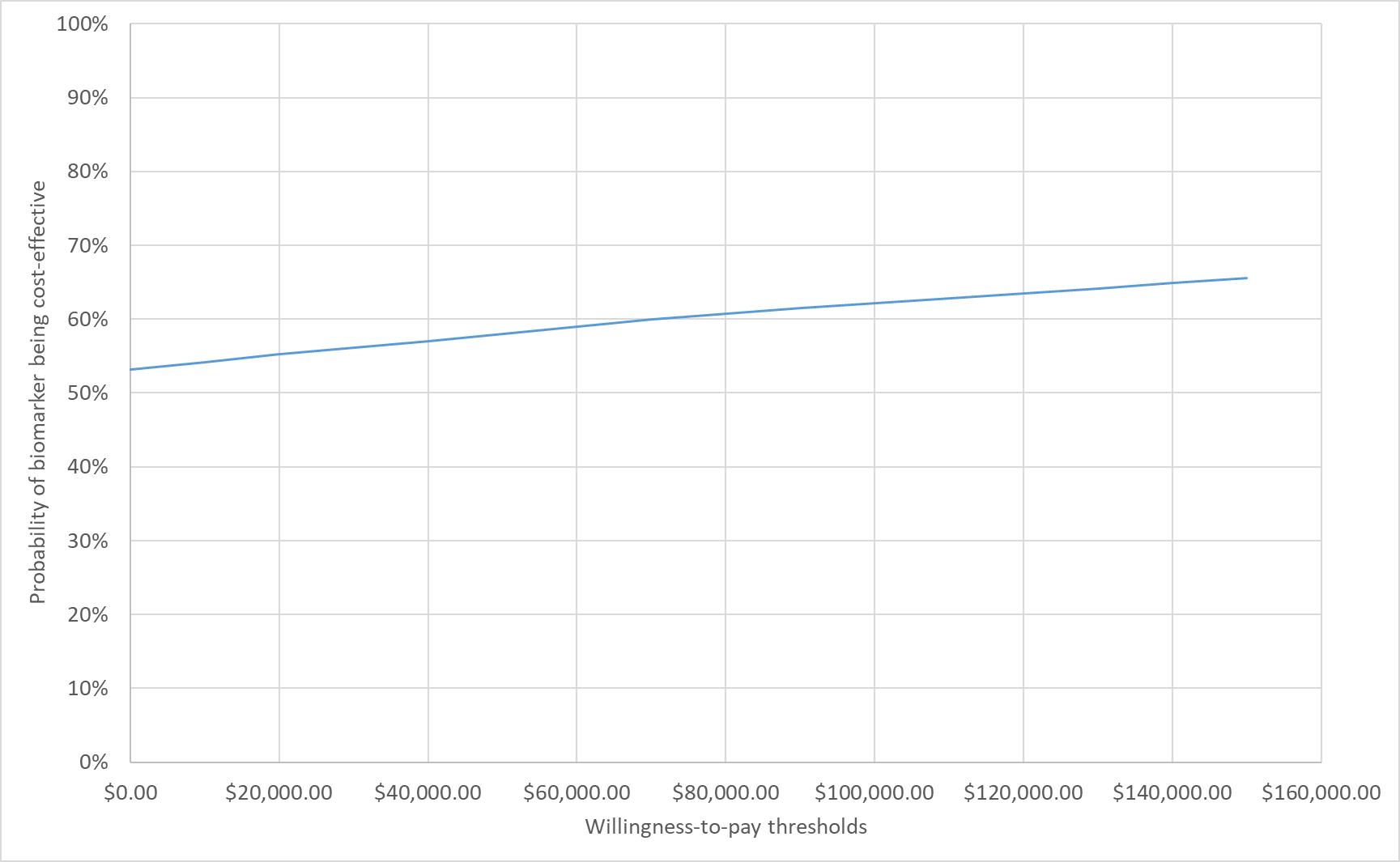


# Figure S3. Cost-effectiveness acceptability curve showing the probability that the biomarker is cost-effective at various willingness-to-pay thresholds.

# REFERENCES

1. Villani AP, Rouzaud M, Sevrain M, Barnetche T, Paul C, Richard MA, et al. Prevalence of undiagnosed psoriatic arthritis among psoriasis patients: Systematic review and meta-analysis. Journal of the American Academy of Dermatology. 2015 Aug 1;73(2):242–8.

2. Reich K, Krüger K, Mössner R, Augustin M. Epidemiology and clinical pattern of psoriatic arthritis in Germany: a prospective interdisciplinary epidemiological study of 1511 patients with plaque-type psoriasis. Br J Dermatol. 2009 May;160(5):1040–7.

3. Iragorri N, Hazlewood G, Manns B, Bojke L, Spackman E, Early Detection to Improve Outcome in People With Undiagnosed Psoriatic Arthritis Study Group. Model to Determine the Cost-Effectiveness of Screening Psoriasis Patients for Psoriatic Arthritis. Arthritis Care & Research. 2021;73(2):266–74.

4. Zaghloul SS, Goodfield MJD. Objective Assessment of Compliance With Psoriasis Treatment. Archives of Dermatology. 2004 Apr 1;140(4):408–14.
